# Morphological Diversity of *Epichloë sinensis* from *Festuca sinensis* Germplasm on the Qinghai–Tibet Plateau

**DOI:** 10.3390/jof12030166

**Published:** 2026-02-25

**Authors:** Junying Liu, Jiawen Sun, Yanqun Zhao, Zhongxiang Li, Mei Zhang, Longxuan Cui, Jinhui Shen, Yang Luo, Yue Gao, Wei Zhou, Taixiang Chen, Tian Wang, Mingxiang Du, Wencong Liu, Chao Xia, Tao Hu, Pei Tian

**Affiliations:** State Key Laboratory of Herbage Improvement and Grassland Argo-Ecosystems, Key Laboratory of Grassland Livestock Industry Innovation, Ministry of Agriculture and Rural Affairs, Engineering Research Center of Grassland Industry, Ministry of Education, College of Pastoral Agriculture Science and Technology, Lanzhou University, Lanzhou 730020, China; jyliu2021@lzu.edu.cn (J.L.); sjw0609@126.com (J.S.); 220220902400@lzu.edu.cn (Y.Z.); 220220902451@lzu.edu.cn (Z.L.); 220220901640@lzu.edu.cn (M.Z.); cuilx2023@lzu.edu.cn (L.C.); shjinhui2023@lzu.edu.cn (J.S.); luoy_lzu@163.com (Y.L.); gaoy2023@lzu.edu.cn (Y.G.); zhouw2023@lzu.edu.cn (W.Z.); chentx@lzu.edu.cn (T.C.); wangt_lzu@163.com (T.W.); dumx2025@lzu.edu.cn (M.D.); 220220902320@lzu.edu.cn (W.L.); xiac@lzu.edu.cn (C.X.); hut@lzu.edu.cn (T.H.)

**Keywords:** altitude, colony, correlation, growth rate, precipitation, spores, temperature

## Abstract

*Epichloë sinensis* engages in mutualistic symbiosis with *Festuca sinensis* on the Qinghai–Tibet Plateau. The influence of variation within the *Epichloë* genus on morphology in this context is poorly understood, as is the influence of environmental factors (e.g., temperature, precipitation, and altitude). Accordingly, a total of 122 fungal endophyte strains were isolated from 270 *F. sinensis* seeds collected from different locations on the Qinghai–Tibet Plateau, and their morphological characteristics were observed. The colonies were white on the front, dark brown in the center on the back, and light brown or yellow around the PDA medium, exhibiting typical characteristics of *E. sinensis*. Morphological diversity was categorized into (1) colony features (six types based on texture, shape, and cracks), (2) growth rates (51 strains that produce spores: 0.23–0.78 mm/d; 71 strains that do not produce spores: 0.11–0.93 mm/d), and (3) hyphal width (51 strains that produce spores: 0.60–2.57 μm; 71 strains that do not produce spores: 0.95–2.10 μm). Correlation analyses revealed that temperature and altitude had significant effects on these traits. Phylogenetic relationships showed that 17 strains probably were *E. sinensis*, and only 4 strains probably were the endophyte *E. poae*. One strain was haploid and may have originated from *E. festucae*. All 22 tested strains lacked genes associated with toxic alkaloid biosynthesis (ergot alkaloid) but harbored regulatory genes for the insect-resistant alkaloid peramine, demonstrating potential for use in developing new germplasm in *Festuca* species.

## 1. Introduction

*Epichloë sinensis* can enhance plant growth and improve the stress resistance of its host grass, namely, *Festuca sinensis* [1,2]. Over 300 species of Poaceae grasses from 80 genera have been found to harbor 51 species of *Epichloë* endophytes [3,4,5]. *Epichloë* species can produce alkaloids that deter, kill, or slow the development of invertebrate herbivores and, in some cases, negatively affect vertebrates as well. Four classes of *Epichloë* alkaloids have been studied extensively regarding biological effects, biosynthetic pathways, genetics, evolution, and variation within each class. These are the ergot alkaloids pyrrolopyrazines, including peramine; indole-diterpenes; and 1-aminopyrrolizidines, including lolines [6].

*Epichloë* endophytes exhibit varying growth rates, colony shapes, and conidia under in vitro conditions. For example, *E. gansuensis* grows symbiotically with *Achnatherum inebrians* at a growth rate of 1.07 mm/d on PDA. Some endophytes, like *E. festucae var. lolii* and *E. coenophialum*, engage in symbiotic relationships with different grasses, such as *Lolium perenne* and *F. arundinacea*, at a slow growth rate (less than 0.57 mm/d). *E. typinum*, which grows symbiotically with *Holcus mollis*, and *E. chilense*, which grows symbiotically with *Dactylis glomerata*, have a relatively fast growth rate of 1.43 mm/d [7]. *E. funki* isolated from *Melica* produces the largest spores (6.5 μm~7.3 μm × 2.8 μm~3.3 μm), while *E. siegelii* isolated from *Achnatherum sibiricum* has smaller spores (3.2 μm~4.5 μm × 2.6 μm~2.8 μm) [8,9].

The growth rate, colony shape, and spore morphology of different strains of the same fungus can differ significantly [10]. A total of 484 fungal endophyte strains were isolated from different geographical populations of *A. sibiricum* in Inner Mongolia and divided into five categories based on morphological characteristics, with one category featuring the slowest growth rate. The growth rates of two other types were moderate but still significantly faster than those of the other three types [11]. Similarly, twenty-two fungal endophyte strains isolated from geographically distinct populations of *Roegneria* spp. were categorized into four morphotypes based on colony morphology. Two morphotypes demonstrated significantly higher growth rates than the other two [12]. A total of 96 fungal endophyte isolates were obtained from three field populations of *Leymus chinensis*. Based on these morphological characteristics, the strains were categorized into three distinct types [13].

*Festuca sinensis* is a high-quality forage grass widely distributed across the Qinghai–Tibet Plateau, exhibiting exceptional stress tolerance, palatability, and nutritional value [14]. *F. sinensis* is frequently symbiotic with *Epichloë sinensis*. Previous studies have isolated and preliminarily characterized *Epichloë* endophytes from *F. sinensis*. For instance, the seeds collected by Yang et al. [15] from two sites (Ganji and Sangke Grasslands) in Gannan, Gansu, are geographically close. Since the fungal endophytes are derived from different individuals of the same site, it is not possible to compare the differences in the fungi carried by plants across different regions. In contrast, Xu [16] isolated 41 *Epichloë* endophyte strains from *F. sinensis* collected across 12 geographical locations in Qinghai, Gansu, and Sichuan provinces; five samples all originated from Ping’an County in Qinghai, which is also geographically close. These samples also represent different individuals from the same site, showing differences in fungal endophytes among the collected seeds. However, the geographic locations in which the samples were collected in these studies were relatively small. Our team collected and preserved over 300 samples of *F. sinensis*, most of which were infected by fungal endophytes. It is worth exploring whether they carry different fungal endophytes and exhibit morphological and functional diversity.

The growth characteristics of fungal endophytes isolated from a host under in vitro conditions may be influenced by the host’s growth environment, including with respect to factors such as altitude. *E. bronicola* strains isolated from regions below an altitude of 3000 m grew faster than strains isolated from regions above 3000 m [17]. The hyphal density and infection rate for fungal endophytes in *E. bronicola* significantly decreased with altitude [18].

Therefore, in this study, we aimed to determine (i) whether all fungal endophytes associated with *F. sinensis* germplasm resources collected from the Qinghai–Tibet Plateau belong to the same species and (ii) whether there are morphological differences between these coexisting endophytes and to (iii) analyze the effects of host habitat parameters (temperature, precipitation, and altitude) on the species categories and morphologies of coexisting endophytes. We isolated fungal endophytes from hundreds of *F. sinensis* samples collected from different geographical populations on the Qinghai–Tibet Plateau, compared their growth and morphological differences, and examined the effects of fungal endophytes and environmental factors (temperature, precipitation, and altitude) on morphological diversity.

## 2. Materials and Methods

### 2.1. Experimental Materials

Our research utilized *F. sinensis* seeds collected across 326 townships in Qinghai (7 cities, 216 townships), Gansu (3 cities, 68 townships), and Sichuan (2 cities, 42 townships) during 13 August to 29 September 2023 field surveys on the Qinghai–Tibet Plateau (collected by Tian et al.). From these 348 germplasms, 122 geographically representative samples were selected for analysis (Supplementary Table S1), covering 80 townships in Qinghai, 12 in Gansu, and 14 in Sichuan. Latitude, longitude, and altitude were recorded at all sampling points. The China Meteorological Center (http://data.cma.cn (accessed on 5 July 2024)) provided daily temperature and precipitation data from weather stations in the province where the sampling site was located. Using the latitude/longitude coordinates of each sampling site, we applied Anusplin interpolation to derive four key climatic variables for the 2013–2022 period (annual mean temperature, mean growing season temperature, total annual precipitation, and total growing-season precipitation).

### 2.2. Isolation of Fungal Endophytes

Fungal endophytes were isolated from healthy (not moldy and plump) seeds. First, the surfaces of the seeds were disinfected (with 70% absolute ethanol for 3 min and 5% sodium hypochlorite solution for 3 min) and then washed three times with sterile distilled water. Next, the seeds were completely dried with sterilized filter paper, placed on PDA (potato dextrose agar) with 100 µg/mL of ampicillin and 50 µg/mL of streptomycin sulfate, sealed with parafilm, cultured in the dark at 22 °C, and examined regularly for endophyte growth for up to 2 weeks. Endophyte colonies growing out from tissues were transferred to fresh media, and all isolates were deposited in the Germplasm Bank of the Center for Grassland Microbiome, Lanzhou University, Lanzhou, China.

### 2.3. Determination of Colony Growth Rate

Colony diameter was measured every 7 days using the cross method, and the colony morphology was observed. Eight replicates were set for each strain and treatment and observed for a total of six weeks. The growth rate of fungal endophyte colonies was calculated using the following formulas [19]:

(1) CGR=(SWCD−IBCD)/CDCD

where CGR is colony growth rate; SWCD is sixth-week colony diameter; IBCD is initial fungus colony diameter; and CDCD is colony diameter cultivation days.

### 2.4. Morphological Observation of Fungal Endophyte Strains

Fungal endophytes were cultivated in the dark at 25 °C with five replicates per strain. Using cultures grown for four weeks, we measured the size of the conidia (width and length, *n* = 50), along with the conidiogenous cells (length, *n* = 50), with an automated upright fluorescence microscope (Olympus Corporation, Tokyo, Japan, Olympus, BX63) [7].

### 2.5. DNA Extraction, Endophyte Detection and Characterization by Multiplex PCR

Total DNA extraction from the strain was performed using a kit from OMEGA (Beijing, China), and the extracted DNA was stored at −20 °C. DNA was extracted from the mycelia of fungal endophytes and analyzed using multiplex polymerase chain reaction (PCR) for *Epichloë* genes encoding translation elongation factor 1-α (*tefA*), β-tubulin (*tubB*), and enzymes for the first steps in the biosynthesis of loline alkaloids, ergot alkaloids, indole-diterpenes, and pyrrolopyrazines using primer sets and methods described in the studies by Moon et al. [20], Zhu et al. [13], and Wang et al. [21] (Supplementary Tables S2 and S3). The PCR cycling conditions were an initial denaturation step of 5 min at 94 °C, followed by 30 cycles of denaturation at 94 °C for 30 s, annealing for 45 s at 45 °C (*tubB*) and 55 °C (*tefA*), a 72 °C extension for 1 min, and a final step at 72 °C for 10 min, before storage at 4 °C. The PCR products were cloned into the TA cloning vector (Sangon Biotech Co., Ltd., Shanghai, China), and 15 to 30 clones were randomly sequenced.

### 2.6. Phylogenetic Analysis

The sequencing results were entered into GenBank (https://www.ncbi.nlm.nih.gov (accessed on 5 January 2025)) to determine whether they belonged to the genus *Epichloë*. Multiple sequence alignment was conducted in MAFFT 7.507 [22]. Conserved blocks were selected using the Gblocks integrated in PhyloSuite v.1.2.2 [23]. The maximum likelihood (ML) phylogenies were reconstructed using IQTREE 2.0.2 [24]. The resulting trees from the ML analyses were graphically edited in iTol (https://itol.embl.de/ (accessed on 30 September 2025)). These sequences were deposited in GenBank under the accession number PX761513-PX761596 (Supplementary Table S4). The housekeeping genes *tubB* and *tefA* were used to construct a phylogenetic tree. The *tefA* sequence of *Claviceps purpurea* (GenBank accession number AF276508) [20] and the *tubB* sequence of *C. purpurea* (GenBank accession number AF062646) [25] were used as the outgroup of the tree.

### 2.7. Data Analysis

Microsoft Excel 2016 (Microsoft Corp., Redmond, WA, USA) was used for data processing. SPSS 18.0 (SPSS Inc., Chicago, IL, USA) and DPS 8.0 (Data Processing System, Zhejiang University, Zhejiang, China) were used for statistical analyses. All 122 strains were consistently classified into two subgroups based solely on sporulation capability: G1: Sporulating strains (*n* = 51); G2: Non-sporulating strains (*n* = 71). The strains were classified into three subclasses based on spore morphology: S1, S2, and S3. One-way ANOVA and multiple comparisons using Duncan’s method were also employed. The measured data were expressed as means ± standard deviations. The morphologies of conidia and conidiophores were analyzed in OriginPro 2025b (OriginLab Corporation, Northampton, MA, USA). Pearson correlation coefficients were calculated to analyze the relationship between mycelial growth rate and climatic conditions at the host-plant collection sites.

## 3. Results

### 3.1. Colony Morphology

Among 348 *F. sinensis* germplasms, the germplasms with few seeds were excluded. Fungal endophytes were isolated from 122/270 collections tested. We attempted to isolate fungal endophytes from more than 100 other samples of *F. sinensis*, but these efforts were unsuccessful because of contamination by microbial species or the slow growth of the fungal endophytes. The 122 fungal endophyte colonies were white at the front, dark brown at the center of the back portion, and light brown or yellow around the midsection. They grew slowly and had typical characteristics of fungal endophytes in the genus *Epichloë*, but they exhibited some differences in texture, color, and other aspects. We divided the colonies into six morphological subclasses (T1, T2, T3, T4, T5, and T6) based on the morphology of their obverse and reverse sides. There was one strain in subclass T1, with a white cotton colony at the front and a tight texture. The center of the colony was moist, while the center of the colony on the back was dark brown with yellow surroundings (Supplementary Figure S1). There were two strains in subclass T2, with white, cotton-like colonies at the front; neat edges; a loose texture; short aerial hyphae; growth close to the culture medium; dark-brown coloration in the center; and yellow coloration at the back (Supplementary Figure S2). There were four strains in subclass T3, with white, cotton-like colonies at the front; a dense texture; a raised or slightly wrinkled area in the center; and yellow-brown coloration at the back (Supplementary Figure S3). There were 89 strains in subclass T4, with white, cotton-like colonies at the front; raised features overall; well-developed aerial hyphae; cracked colonies at the back; dark-brown coloration in the center; and light-brown or yellow coloration on the sides (Supplementary Figure S4). There were 25 strains in subclass T5, with white, cotton-like colonies at the front; a dense texture; a central bulge; irregular edges; cracked colonies at the back; dark-brown coloration in the center; and light-brown or yellow coloration around the sides (Supplementary Figure S5). There was one strain in subclass T6, with white and fluffy or cotton-like colonies at the front, a raised or slightly wrinkled area in the center, short aerial hyphae, cracked colonies at the back, a dark-brown area in the center, and light-brown or yellow coloration on the sides (Supplementary Figure S6).

### 3.2. Growth Rate

**Figure 1:**
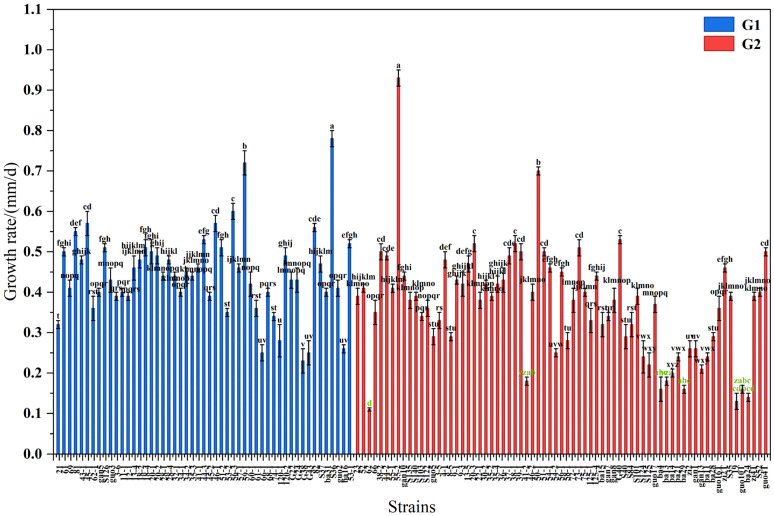
Growth rate of *Epichloë* fungal endophytes. Notes: Subclass G1 denotes growth rates of sporulating strains, while G2 represents those of non-sporulating strains. Different lowercase letters indicate significant differences in fungal endophyte strains within the same group (*p* < 0.05). The green color indicates the significance of strains with slower growth rates.

The colony growth rates of 122 strains (Figure 1), which ranged from 0.14 mm/d to 0.93 mm/d, were divided into two subclasses based on whether they produced spores. There were 51 strains producing spores in subclass G1, ranging from 0.23 mm/d to 0.78 mm/d. Strain S36 had a significantly higher growth rate than strains 59-1 and 56-3 (*p* < 0.05), ranging from 0.60 mm/d to 0.78 mm/d. Strains 120-1, ba16, 66-1, G43, and G38 had slower growth rates, ranging from 0.23 mm/d to 0.28 mm/d. There were 71 strains not producing spores in subclass G2, ranging from 0.11 mm/d to 0.93 mm/d. Strain 55-2 had a significantly higher growth rate than strain 50-1 (*p* < 0.05), ranging from 0.70 mm/d to 0.93 mm/d.

### 3.3. Hyphal Width

**Figure 2:**
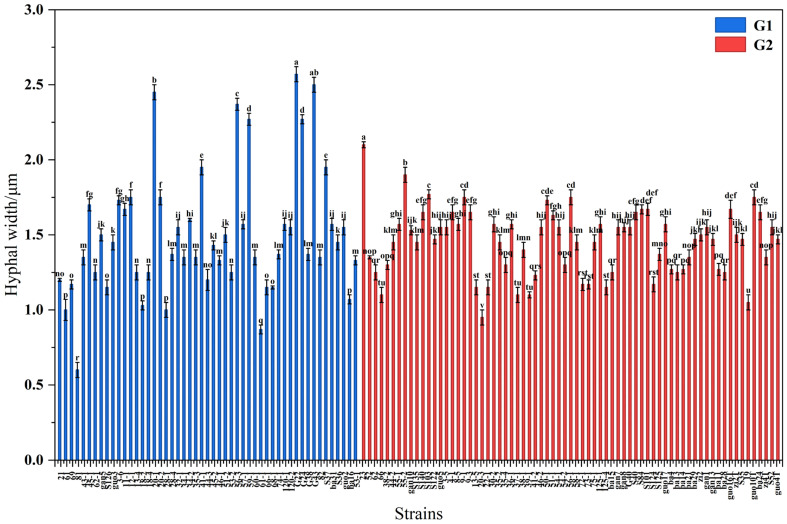
Mycelial width of *Epichloë* fungal endophytes. Notes: Subclass G1 denotes growth rates of sporulating strains, while G2 represents those of non-sporulating strains. Different lowercase letters indicate significant differences in fungal endophyte strains within the same group (*p* < 0.05).

The colony hyphae width of 122 strains (Figure 2), ranging from 0.60 μm to 2.50 μm, was divided into two subclasses based on whether they produced spores. There were 51 spore-producing strains in subclass G1, ranging from 0.60 μm to 2.57 μm. Strains G22 and G43 had significantly wider hyphae than strains 20-1, 56-3, 59-1, G24, 41-1, and S37 (*p* < 0.05), ranging from 1.95 μm to 2.57 μm. Strain 81 had the narrowest hyphae. There were 71 non-spore-producing strains in subclass G2, ranging from 0.95 μm to 2.10 μm. Strain 23 had significantly wider hyphae than strain 55-2 (*p* < 0.05), ranging from 1.90 μm to 2.10 μm. Strain 20-3 had the narrowest hyphae.

### 3.4. Length and Width of Spores

**Figure 3:**
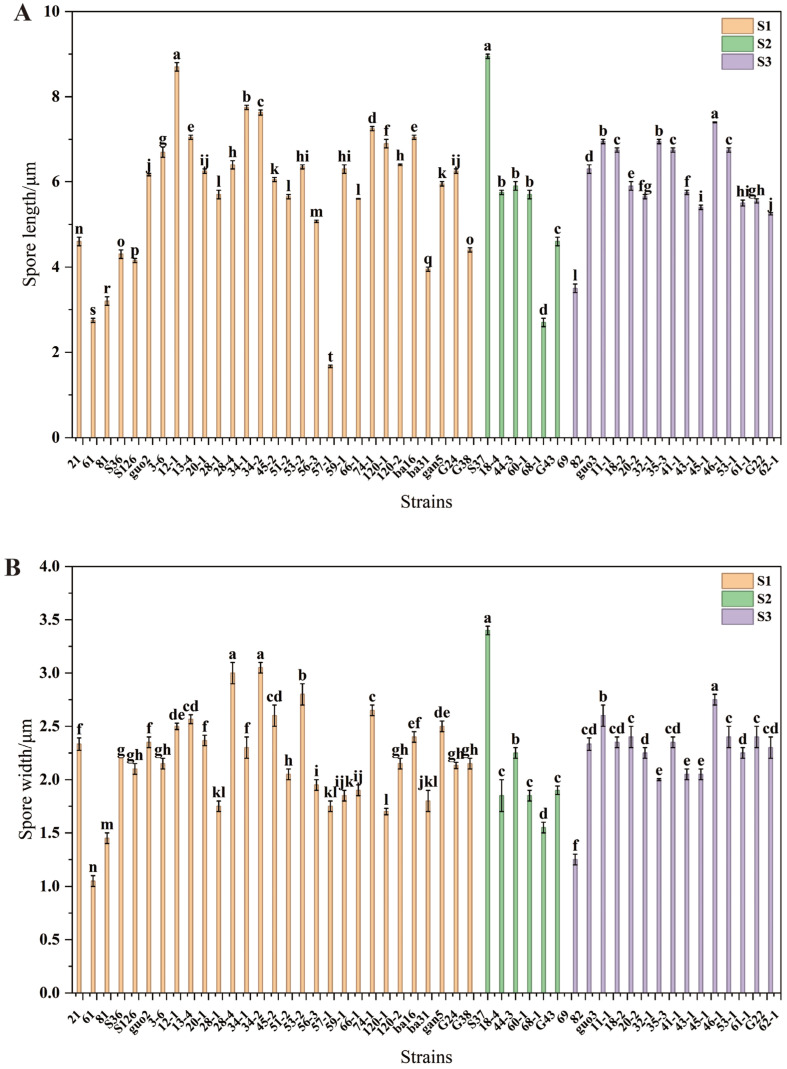
(**A**) Spores length of *Epichloë* fungal endophytes; (**B**) Spores width of *Epichloë* fungal endophytes. Notes: Orange pillar: Spores in subclass S1 exhibit a crescent-shaped morphology. Green pillar: Spores in subclass S2 are elliptical. Purple pillar: Spores in subclass S3 are crescent-shaped or elliptical. Different lowercase letters indicate significant differences in fungal endophyte strains within the same group (*p* < 0.05). The same applies below.

Among the 122 fungal endophytes, 51 strains produced spores, whereas 71 strains did not. The strains were classified into three subclasses based on spore morphology: S1, S2, and S3 (Supplementary Figure S7). There were 29 strains in subclass S1, and their spores were crescent-shaped. Six subclass S2 strains exhibited elliptical spores. There were 16 S3 subclass strains with crescent or oval spores. The length of the conidia ranged between 1.67 μm and 8.95 μm (Figure 3A). In subclass S1, the length of the conidia ranged between 1.67 μm and 8.70 μm. Relative to the other strains, strain 12-1 had significantly longer conidia (*p* < 0.05), whereas strain 57-1 had significantly shorter conidia (*p* < 0.05). In subclass S2, the length of the spores ranged from 2.70 to 8.95 μm. Relative to the other strains, strain S37 had significantly longer spores (*p* < 0.05), and strain 68-1 had significantly shorter spores (*p* < 0.05). In subclass S3, spore length ranged between 3.50 μm and 7.40 μm. Relative to the other strains, strain 45-1 had significantly longer spores (*p* < 0.05), whereas strain 69 had significantly shorter spores (*p* < 0.05).

The width of the spores ranged from 1.05 μm to 3.40 μm (Figure 3B). In subclass S1, the width of the spores ranged from 1.05 μm to 3.00 μm. Relative to the other strains, strain 28-4 had significantly wider spores (*p* < 0.05), whereas strain 61 had significantly narrower spores (*p* < 0.05). In subclass S2, spore width ranged between 1.55 μm and 3.40 μm. Relative to the other strains, strain S37 had significantly wider spores (*p* < 0.05), whereas strain 68-1 had significantly narrower spores (*p* < 0.05). In subclass S3, the width of the spores ranged from 1.25 μm to 2.75 μm. Relative to the other strains, strain 45-1 had significantly wider spores (*p* < 0.05), whereas strain 69 had significantly narrower spores (*p* < 0.05).

### 3.5. Length, Top Width, and Base Width of Conidiophores

**Figure 4:**
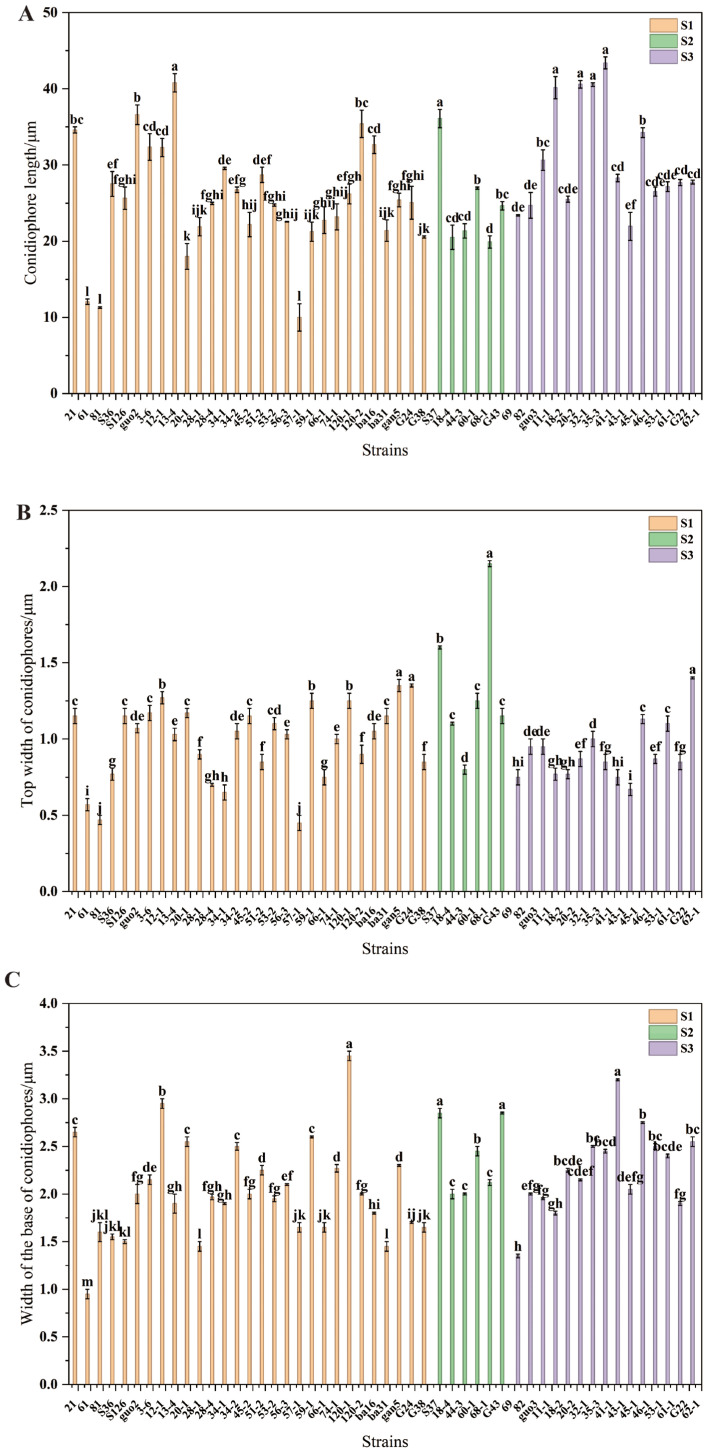
(**A**) The length of the conidiophores of *Epichloë* fungal endophytes; (**B**) The top width of the conidiophores of *Epichloë* fungal endophytes; (**C**) The base width of the conidiophores of *Epichloë* fungal endophytes.

The length of the conidiophores ranged between 10.00 μm and 43.40 μm (Figure 4A). In subclass S1, the length of conidiophores ranged between 10.00 μm and 40.80 μm. Relative to the other strains, strain 13-4 had significantly longer conidiophores (*p* < 0.05), while strain 57-1 had significantly shorter conidiophores (*p* < 0.05). In subclass S2, the length of conidiophores ranged between 19.9 μm and 36.10 μm. Relative to the other strains, strain S37 had significantly more conidiophores (*p* < 0.05), while strain 68-1 had significantly fewer conidiophores (*p* < 0.05). In subclass S3, the length of the conidiophores ranged from 21.95 to 43.40 μm. Relative to the other strains, strains 35-3, 20-2, and 32-1 had significantly longer conidiophores (*p* < 0.05), while strain 62-1 had significantly shorter conidiophores (*p* < 0.05).

The top width of the conidiophores ranged between 0.45 μm and 2.15 μm (Figure 4B). In subclass S1, the top width of the conidiophores ranged from 0.45 μm to 1.35 μm. Relative to the other strains, the top widths of the conidiophores of strains gan5 and G24 were significantly greater (*p* < 0.05), whereas the top widths of the conidiophores of strain 57-1 were significantly lower (*p* < 0.05). In subclass S2, the top widths of the conidiophores ranged from 0.80 μm to 2.15 μm. The top widths of the conidiophores of strain 68-1 were significantly greater than those of the other strains (*p* < 0.05), whereas the top widths of the conidiophores of strain 44-3 were significantly lower than those of the other strains (*p* < 0.05). In subclass S3, the top width of the conidiophores ranged from 0.67 to 1.40 μm. Strain G22 exhibited significantly greater top width of its conidiophores than the other strains (*p* < 0.05), while strain 43-1 had significantly lower top width of its conidiophores relative to the other strains (*p* < 0.05).

The base width of the conidiophores ranged between 0.95 μm and 3.45 μm (Figure 4C). In subclass S1, the base width of the conidiophores ranged from 0.95 μm to 3.45 μm. The conidiophore base width for strain 120-1 was significantly greater than that of the other strains (*p* < 0.05), whereas the conidiophore base widths of strain 61 were significantly lower than those of the other strains (*p* < 0.05). In subclass S2, the width range of the conidiophore base was 2.00–2.85 μm. The conidiophore base widths of strains S37 and G43 were significantly greater than those of the other strains (*p* < 0.05), whereas the conidiophore base widths of strains 18-4, 44-3, and 68-1 were significantly lower relative to the other strains (*p* < 0.05). In subclass S3, the width range of the conidiophore base was between 1.35 μm and 3.20 μm. The conidiophore base width of strain 41-1 was significantly greater than that of the other strains (*p* < 0.05), whereas the conidiophore base width of strain 69 was significantly lower than that of the other strains (*p* < 0.05).

### 3.6. Principal Component Analysis (PCA)

**Figure 5:**
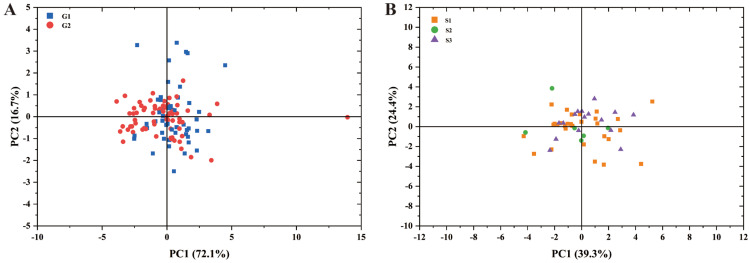
Principal component analysis of the morphological diversity of *Epichloë* fungal endophytes. Note: (**A**) is a principal component analysis based on the hyphal width of 122 endophytes, colony diameter, and the growth rate of the strains. The fungal endophytes in group G1 were sporulating strains, while those in group G2 were non-sporulating strains. (**B**) is a principal component analysis based on the colony diameter, growth rate, hyphal width, top width of conidiophores, base width of conidiophores, and size of the conidia of 51 fungal endophyte strains that had already produced spores. Regarding morphology, the spores in groups S1, S2, and S3 were crescent-shaped, elliptical, and crescent-shaped or elliptical, respectively.

Based on whether the strains produce spores, 122 fungal endophytes were divided into two groups. Principal component analysis (PCA) was then performed using three key morphological traits: hyphal width, colony diameter, and growth rate (Figure 5A). The first principal component explained approximately 72.1% of the variability, whereas the second principal component explained 16.7%. There was no significant complete separation between groups G1 and G2.

The strains were classified into three groups based on spore morphology: S1, S2, and S3. PCA was conducted on the colony diameter, growth rate, hyphal width, top and base widths of conidiophores, and size of the conidia of 51 fungal endophyte strains that had already produced spores (Figure 5B). The first principal component explained approximately 39.3% of the variability, whereas the second principal component explained 24.4%. There was no significant complete separation between groups S1, S2, and S3.

### 3.7. Correlation Analysis Between Various Indicators of F. sinensis and Climatic Characteristics

**Figure 6:**
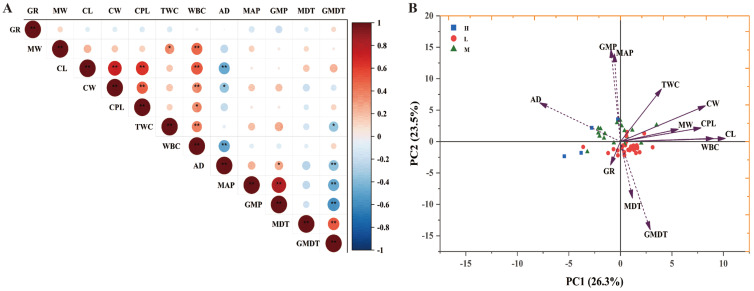
Correlation analysis (**A**) and principal component analysis (**B**) of fungal endophyte hyphal width, growth rate, spore size, and conidiophore size with respect to the climate and environment of the host collection site. Note: * and ** indicate significant correlations at *p* < 0.05 and *p* < 0.01. The value is the correlation coefficient, P. Abbreviations: Growth rate (GR), mycelial width (MW), conidium length (CL), conidium width (CW), conidiophore length (CPL), top width of a conidiophore (TWC), width of the base of a conidiophore (WBC), altitude (AD), annual average precipitation (MAP), precipitation during the growing season (GMP), annual mean temperature (MDT), and average temperature during the growing season (GMDT). H represents fungal endophytes isolated from *F. sinensis* collected at an altitude of 4000~5100 m. L represents fungal endophytes isolated from *F. sinensis* at an altitude of 2000~3000 m. M represents fungal endophytes isolated from *F. sinensis* at an altitude of 3000~4000 m.

The length of the conidia and width of the conidia stem bases of 51 spore-producing strains were significantly negatively correlated with altitude (*p* < 0.01). Conidium width was significantly negatively correlated with altitude (*p* < 0.05; Figure 6A). Based on the results of the correlation analysis, we further grouped the fungal endophyte strains at different altitudes for PCA of morphological indicators (Figure 6B). The first principal component (PC1) explains approximately 26.3% of the variability, while the second principal component (PC2) explains 23.5%. Fungal endophytes isolated from *F. sinensis* collected at an altitude of 3000–4000 m (M) were distributed on the positive axis of PC2, while the fungal endophytes isolated from *F. sinensis* collected at an altitude of 2000–3000 m (L) were distributed on the negative axis. The length, width, top width, and base width of conidia were distributed along the positive axis of PC1 and closely related to whether fungal endophytes were grown at altitudes of 2000–3000 m (L) and 3000–4000 m (M). Growth rate was distributed in the third quadrant and closely related to whether fungal endophytes were grown at altitudes of 2000–3000 m (L).

### 3.8. tef-α and tubB Amplification in E. sinensis

**Figure 7:**
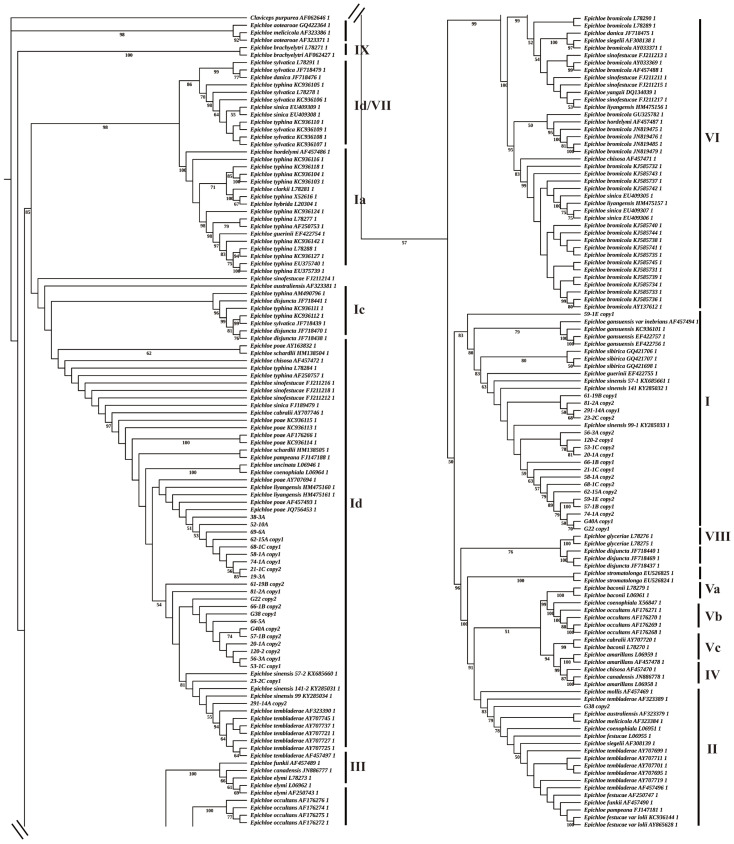
A Maximum Likelihood (ML) phylogenetic tree constructed based on partial sequences of the *tubB* gene.

A total of 22 fungal endophyte strains with significant altitude and temperature differences were selected for sequencing. After constructing a phylogenetic tree based on partial sequences of *tef*-*α* (Supplementary Figure S8) and *tubB* (Figure 7). We identified 17 strains that probably were *E. sinensis*. Two copies were obtained from the *E. poae* clade (Ib) and the *E. sibirica* clade (I), respectively, while strains 19-3A, 52-10A, 66-5A, and 69-6A probably were the endophyte *E. poae*. Additionally, G38 was haploid and may originate from *E. festucae.*

### 3.9. Mating Types and Alkaloid Gene Profiling

To preliminarily evaluate the alkaloid production characteristics of the fungal endophytes, we used PCR to amplify 39 regulatory genes of four major alkaloid biosynthesis pathways from the isolated and identified fungal endophytes (Table 1, Table 2, Table 3 and Table 4). A total of 21 strains tested had 1–2 genes in the loline alkaloid biosynthesis pathway and may not produce loline alkaloids (Table 1). Strain 19-3A contains all seven genes in the loline alkaloid biosynthesis pathway (*lolM*, *lolA*, *lolC*, *lolU*, *lolF*, *lolD*, and *lolT*) and predicted to produce loline alkaloids. Several genes were involved in the biosynthesis pathway for peramine alkaloids, which can produce peramine alkaloids (Table 2). All strains possess some genes involved in the indole-diterpenoid biosynthesis pathway but do not produce lolitrem B (Table 3). There was a lack of genes related to the ergot alkaloid biosynthesis pathway (Table 4). This result was similar to previously reported results regarding an *E. sinensis* model strain [26]. Two strains (ID: 19-3A, G38) only contain the mating type gene *mtAC*, belonging to mating type A (Table 5). A total of 20 strains contain both *mtAC* and *mtBA*, belonging to mating type AB.

## 4. Discussion

### 4.1. Diversity of E. sinensis

*Epichloë* endophytes display substantial morphological diversity [27]. In this study, fungal endophytes isolated from 122 *F. sinensis* germplasms showed significant phenotypic variation in terms of growth rate, conidial morphology, and colony diameter. However, molecular evidence suggests these variants likely belong to *E. sinensis*, a recently described species. This phenotypic divergence may reflect (i) host–endophyte coevolutionary adaptations specific to *F. sinensis* ecotypes or (ii) genetic polymorphism among fungal strains, potentially driven by geographical isolation [28]. In previous studies, *Echinopogon ovatus* endophytes were classified into two morphotypes based on growth rate and hyphal curvature [20]. Type 1 exhibits a slow growth rate and curved hyphae, encompassing all New Zealand populations plus two Australian populations. The colonies in New Zealand populations are predominantly brown, while Australian populations appear slightly yellow. Type 2 fungal populations display a fast growth rate with a felt-like colony surface capable of conidium production, comprising two additional Australian populations [20]. In our study, we identified six distinct fungal endophyte morphotypes associated with *F. sinensis*. This morphological diversity suggests that nutrient availability and intercellular space dimensions within host tissues may drive phenotypic plasticity in these endophytes [26]. Competitive interactions among co-occurring endophytes are likely key drivers of phenotypic diversification, enabling niche partitioning within hosts [27]. These findings collectively highlight the morphological plasticity of *F. sinensis*-associated endophytes. We speculate that this mainly results from genetic differences among *E. sinensis* strains and long-term selection pressures affecting different *F. sinensis* ecotypes, which drive adaptive differentiation and influence both ecological adaptability and the stability of symbiotic relationships.

Nutritional circumstances and genotypic divergence are the main factors influencing fungal endophytes’ growth features [29]. Geographical environmental gradients within conspecific hosts can lead to morphological variation [30]. In this study, growth rates and conidial dimensions (length, width, stem length, and apical/base width) differed significantly among populations (*p* < 0.05). These variations likely reflect host environmental adaptations and endophyte genomic plasticity [31], potentially amplified by polyploidy/hybridization events that drive structural variation during chromosomal rearrangements or transposon-mediated recombination—processes known to alter hyphal, conidial, and sclerotial morphogenesis [32]. Our conidial values are consistent with previous reports regarding conidial lengths (4.8–6.2 μm) and growth rates (0.22–2.86 mm/d) for isolates from Inner Mongolia [33]. North African tall fescue endophytes have the following conidial length distribution: <6.5 μm “short-type” vs. ≥6.5 μm “long-type” [34]. Importantly, this study’s fast-growing strains only produced short-type conidia (4.3 ± 0.1 μm × 2.2 ± 0.1 μm) with wider hyphae. This information suggests that conidial size is correlated with genetic architecture and environmental selection [35].

### 4.2. The Impact of Environmental Factors on the Diversity of E. sinensis

The composition, temperature, light, and humidity of the culture medium under in vitro culture conditions can affect the growth rate and colony morphology of fungal endophyte strains. Research has found that the optimal pH for the growth of fungal endophytes is 4–7, with D-sorbitol serving as the strongest carbon source and peptone serving as the nitrogen source. PDA and PSA media are more suitable for mycelial growth [16]. In this study, identical culture media and conditions were used, and the differences may have originated from the colonies themselves, which are influenced by the host genotype and growth environment. The hosts of the 122 strains isolated in this study were collected from different locations. Their altitudes range from 2630 m to 5197 m, with rainfall varying from 102 to 808 mm and annual average temperatures ranging from −7.5 to 8.5 °C.

In our study, the conidial length of endophytes from colder host sites (with a mean annual temperature of −7.5 °C) significantly exceeded that of strains from warmer sites (8.5 °C). The width of the top of the conidial stem was narrow, and the width of the base of the conidial stem was wide. The conidia were shorter, and the stem bases of the conidia were narrower at high altitudes (4594 m). The temperature at the host’s growth site appears to be a key determinant of fungal endophyte phenotypic expression, influencing colony growth rate, morphology, and sporulation characteristics [35,36]. For instance, in vitro cultures of *Epichloë festucae var. lolii*, *E. coenophiala*, and *E. typina* exhibit optimal growth at 25 °C [37]. Growth rates of *F. sinensis* endophytes diverged significantly across geographical populations under identical temperature regimes. Growth rates under optimal thermal conditions define three phenotypic clusters: fast-, intermediate-, and slow-growing strains [16]. The symbiotic relationship between fungal endophytes and their host grass may be impacted by environmental conditions [1]. The infection rate of fungal endophytes in tall fescue was considerably lower when the average monthly temperature was below the minimal temperature needed for fungal endophyte growth in the United States [37]. Endophyte colonization patterns exhibit distinct environmental dependencies across ecosystems. In Southern Australia, endophyte colonization rates in *Lolium rigidum* show a positive correlation with mean precipitation levels [38]. Polish grassland systems demonstrate an inverse relationship, with colonization rates being negatively associated with precipitation but positively correlated with temperature [39]. Altitudinal gradients reveal consistent declines in both the colonization rates and hyphal density of *Epichloë bromicola*, accompanied by progressive reductions in colony diameter [18]. Growth rates declined significantly with increasing altitude, mirrored by suppressed growth in high-altitude (>3000 m) *Elymus*-associated endophytes relative to low-altitude counterparts [17]. This altitudinal constraint aligns with our findings and likely stems from host-mediated metabolic shifts: plants in distinct environments synthesize secondary metabolites (phenolics, flavonoids, and terpenes) and nutrients (nitrogen, phosphorus, and sugars) differentially. When transferred to an in vitro culture, these compounds directly modulate fungal morphogenesis via cell wall interactions, membrane receptor binding, or signaling cascades, ultimately altering hyphal branching and conidiation patterns [40]. It is evident that the host’s geographic location may have an impact on the growth features of fungal endophytes due to a variety of environmental factors working together [41].

*Epichloë* endophytes are generally hybridized by asexual or sexual fungal endophytes and may possess 2–3 copies of *TubB* and *Tef1-α* or other conserved genes of different origins [42]. In our study, phylogenetic relationships revealed that *E. sinensis* was an asexual interspecific hybrid of *E. poae* and *E. sibirica* based on intron sequences of housekeeping genes for β-tubulin (TUBB) and translation elongation factor 1-*α* (TEF1-*α*). Research has found that tall fescue can carry *E. coenophiala*, *Neotyphodium* sp. FaTG-2, and *Neotyphodium* sp. FaTG-3, FaTG-4, and FaTG-5 simultaneously, while ryegrass can carry *E. festucae* and *E. typina*. *A. inebrians* can carry *E. gansuensis* and *E. inebrians*, which exhibit significant diversity in terms of genotype, molecular markers, and alkaloid-producing genes [7]. This information indicates that hybrid or polyploid hosts can influence endophyte diversity and may facilitate hybridization events. Sexual reproduction is largely confined to haploid, non-hybrid species, whereas asexual transmission predominates in haploid and hybrid heteroploid taxa, with only a few exceptions [43]. The phenomenon of the same host carrying different endophytes provides an important model system for studying the mechanisms of interaction between fungal endophytes and host plants.

Hybrid *Epichloë* species with more copies often have multiple *EAS*, *IDT*, or *LOL* clusters, which could lead to greater benefits for the host grasses, especially in regard to their ability to produce alkaloids [44]. In this study, a total of 22 tested strains lacked genes associated with toxic-alkaloid-regulatory genes (ergot alkaloid), but they did contain the insect-resistant alkaloid polyamine regulatory gene, which can be used for the creation of new germplasms in the genus *Festuca*. A total of 20 strains were categorized as *MtaC* and *MtbA*. Three haploid fungal endophyte strains were found to carry both mating-type genes (*MtaC* and *MtbA*). This phenomenon may result from ancestral hybridization events that led to the retention of mating-type genes from both parental strains in a single nucleus, with incomplete meiotic separation producing haploid progeny containing both types of genes [45]. In addition, this could reflect insufficient clones, necessitating the sequencing of a greater number of clones for verification.

## 5. Conclusions

A total of 122 fungal endophytes were isolated from the germplasm resources of *F. sinensis* collected from the Qinghai–Tibet Plateau. This endophyte exhibits typical morphological characteristics of the *Epichloë* genus. A total of 17 endophyte strains were consistent with those reported for *E. sinensis* and may be the same species, but their growth and morphology are different.

All 22 tested strains lacked genes associated with toxic alkaloid biosynthesis (ergot alkaloid) but harbored regulatory genes for the insect-resistant alkaloid peramine, demonstrating potential for use in developing new germplasm in *Festuca* species.

The size of conidia may be influenced by the temperature and altitude of the host’s growth site.

## Figures and Tables

**Table 1:** Loline biosynthesis pathway regulating gene profiling.

Strain	Total Number	Loline Genes						
		*lolM*	*lolA*	*lolC*	*lolU*	*lolF*	*lolD*	*lolT*
19-3A	1	+	+	+	+	+	+	+
53-1C	1	−	−	+	−	−	−	−
21-1C, 23-2C, 69-6A, 52-10A, 66-5A, 56-3A, 57-1B, 58-1A, 59-1E, 66-1B, 68-1C, 74-1C, 120-2	13	−	−	+	−	−	+	−
61-19B, 81-2A, 62-15A, 20-1A, G40A	5	−	−	−	−	−	+	−
G22, G38	2	−	−	−	−	−	−	−
57A	1		−	+	−	−	−	−

Note: The plus sign (+) indicates that the specific gene fragment was amplified via PCR; the minus sign (−) indicates that the specific gene fragment could not be amplified via PCR. Strain 57 served as a control. The same applies below.

**Table 2:** *ppzA* gene profiling conducted to determine alkaloid chemotype classes.

Strain	Total Number	*ppzA* (Formerly Known as *perA*) Genes							
		*ppzA-A2*	*ppzA-M*	*ppzA-T1*	ppzA-R *	*ppzA-C*	*ppzA-A1*	*ppzA-*∆*R* *	*ppzA-T2*
21-1C	1	+	−	+	+	−	+	−	+
23-2C	1	+	+	+	+	−	+	−	+
61-19B	1	−	+	−	−	−	+	−	+
81-2A	1	−	−	−	−	−	+	−	+
69-6A	1	+	+	−	−	−	−	−	+
52-10A	1	−	+	+	−	−	+	−	+
62-15A	1	−	+	+	−	−	+	−	−
66-5A	1	−	+	−	+	−	+	−	+
G22A	1	−	−	+	−	+	−	−	+
G38	1	−	−	+	−	+	+	−	+
G40A	1	+	−	+	−	+	+	−	+
19-3A, 20-1A, 58-1A, 68-1C	4	+	+	+	+	+	+	−	+
53-1C, 56-3A, 59-1E, 74-1C, 120-2	5	+	+	+	+	+	+	+	+
57-1B, 66-1B	2	−	+	+	+	+	+	−	+
57A	1	+					+		

Note: The plus sign (+) indicates that the specific gene fragment was amplified via PCR; the minus sign (−) indicates that the specific gene fragment could not be amplified via PCR. Strain 57 served as a control. ppzA-R * means *ppzA* from which the R domain was deleted. The *ppzA-*∆R * (representing allele *ppzA-2*) refers to the *ppzA* gene from which the R-domain has been deleted, the functional implication of this deletion is the absence of the ffnal enzymatic step required to convert diketopiperazine into peramine in the ∆R variant, resulting in the production of pyrrolopyrazine-1,4-diones instead of peramine.

**Table 3:** Indole-diterpene gene profiling conducted to determine alkaloid chemotype classes.

Strain	Total Number	Indole-Diterpene Genes										
		*ltmJ*	*ltmE*	*ltmS*	*ltmG*	*ltmF*	*ltmM*	*ltmC*	*ltmP*	*ltmQ*	*ltmB*	*ltmK*
19-3A	1	−	−	+	+	−	*	−	−	−	−	*
21-1C	1	−	−	+	−	−	−	+	−	+	+	+
23-2C	1	−	−	+	+	−	+	+	−	+	+	+
61-19B	1	−	−	+	−	−	+	−	−	−	+	−
81-2A	1	−	−	+	−	−	+	−	−	+	+	+
69-6A	1	−	−	+	−	−	−	+	−	−	+	+
52-10A	1	−	−	+	+	−	+	+	−	+	+	+
62-15A	1	−	−	+	+	−	−	+	−	−	+	+
66-5A	1	−	−	+	+	−	+	+	+	+	+	+
57-1B	1	−	*	+	+	+	−	+	−	+	+	+
66-1B, 120-2	2	−	−	+	+	+	+	+	−	+	+	+
G22A	1	−	−	+	+	−	+	+	−	−	+	+
20-1A, 56-3A, 58-1A, 68-1C, 74-1C, G38, G40A	7	−	−	+	+	+	+	+	+	+	+	+
53-1C, 59-1E	2	−	*	+	+	+	+	+	+	+	+	+
57A	1	−	−		+	+	+	+	+	+	+	+

Note: The plus sign (+) indicates that the specific gene fragment was amplified via PCR; the minus sign (−) indicates that the specific gene fragment could not be amplified via PCR. An asterisk (*) denotes nonspecific amplification. Strain 57 served as a control. The same applies below.

**Table 4:** Ergot alkaloid gene profiling conducted to determine alkaloid chemotype classes.

Strain	Total Number	Ergot Alkaloid Genes												
		*dmaW*	*easA*	*easF*	*easE*	*easD*	*easG*	*easH*	*easO*	*easP*	*cloA*	*lpsB*	*lpsA*	*lpsC*
19-3A	1	+	*	−	*	*	*	*	*	−	−	−	−	−
21-1C	1	+	*	−	*	*	−	*	*	−	−	−	−	−
58-1A, 59-1E, 74-1C	3	−	−	−	−	−	−	+	−	−	−	−	−	−
23-2C, 81-2A, 61-19B, 52-10A, 62-15A, G22, G38, G40, 69-6A, 66-1B, 66-5A, 20-1A, 53-1C, 56-3A, 57-1B, 68-1C, 120-2	17	−	−	−	−	−	−	−	−	−	−	−	−	−
57A	1	−	−	−	−	−	−	−	−	−	−	−	−	−

Note: The plus sign (+) indicates that the specific gene fragment was amplified via PCR; the minus sign (−) indicates that the specific gene fragment could not be amplified via PCR. An asterisk (*) denotes nonspecific amplification. Strain 57 served as a control.

**Table 5:** Mating type genes.

Strain	Total Number	Mating-Type Genes	
		*MtaC*	*MtbA*
19-3A, G38	2	+	−
21-1C, 23-2C, 61-19B, 81-2A, 69-6A, 52-10A, 62-15A, 66-5A, 20-1A, 53-1C, 56-3A, 57-1B, 58-1A, 59-1E, 66-1B, 68-1C, 74-1C, 120-2, G22A, G40A	20	+	+
57A	1	+	+

Note: The plus sign (+) indicates that the specific gene fragment was amplified via PCR; the minus sign (−) indicates that the specific gene fragment could not be amplified via PCR. Strain 57 served as a control.

## Data Availability

All data supporting the findings of this study are available within the paper.

## Supplementary Materials

The following supporting information can be downloaded at: https://www.mdpi.com/article/10.3390/jof12030166/s1. Figure S1. Surface and reverse characteristics of fungi colonies in some strains of T1 subclass. Note: A is surface of ba28, and B is reverse of ba28; Figure S2. Surface and reverse characteristics of fungi colonies in some strains of T2 subclass. Note: A is surface of ba31, B is reverse of ba31, C is surface of 35-4, and D is reverse of 35-4; Figure S3. Surface and reverse of fungi colonies in some strains of T3 subclass. Note: A is surface of zi4T, B is reverse of zi4T, C is surface of gan7, D is reverse of gan7, E is surface of S40, and F is reverse of S40; Figure S4. Surface and reverse characteristics of fungi colonies in some strains of T4 subclass. Note: A is surface of 45-2, B is reverse of 45-2, C is surface of 20-2, D is reverse of 20-2, E is surface of gan5, and F is reverse of gan5; Figure S5. Surface and reverse of fungi colonies in some strains of T5 subclass. Note: A is surface of ba24, B is reverse of ba24, C is surface of guo10T, and D is reverse of guo10T; Figure S6. Surface and reverse characteristics of fungi colonies in some strains of T6 subclass. Note: A is surface of guo4T, and B is reverse of guo4T; Figure S7. The morphology of conidia and conidiophores of some *E. sinensis*. Note: A is strain gan5, with a half moon shaped spore morphology, belonging to the S1 subclass. B is strain 60-1, with elliptical spore morphology, belonging to the S2 subclass. C is strain 46-1, with spore morphology of crescent or oval, belonging to the S3 subclass. D is strain 28-1, with a half moon shaped spore morphology, belonging to the S1 subclass. E is strain S37, with elliptical spore morphology, belonging to the S2 subclass. F is strain 62-1, with spore morphology of crescent or oval, belonging to the S3 subclass; Figure S8. Maximum likelihood phylogenetic tree constructed based on partial sequence of *tef* gene; Table S1. Longitude, latitude, and altitude of sampling points; Table S2. The primer sequence of *TubB* and *Tef1-α* gene amplification; Table S3. Primers used for mating type and alkaloid gene and mating type genes and expected product sizes; Table S4. *TubB* and *Tef1-α* sequences used in phylogenetic analysis in this study.
