# Supplementary material for: Morphological Diversity of Epichloë sinensis from Festuca sinensis Germplasm on the Qinghai–Tibet Plateau
Source: J Fungi (Basel). 2026 Feb 25;12(3):166. doi: 10.3390/jof12030166 (PMC13027652; doi:10.3390/jof12030166)
Supplement: Supplementary file 1 [file jof-12-00166-s001.zip › Supplementary Figure1-9 .pdf]

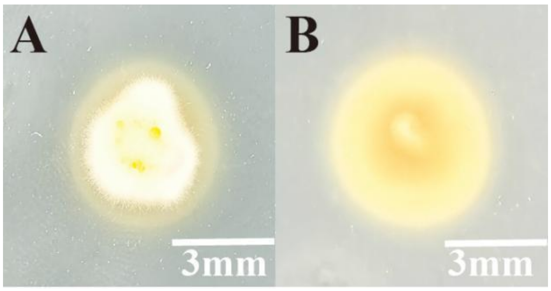

Supplementary Figure S1. Surface and reverse characteristics of fungi colonies in some strains of T1 subclass. Note: A is surface of ba28, and B is reverse of ba28.

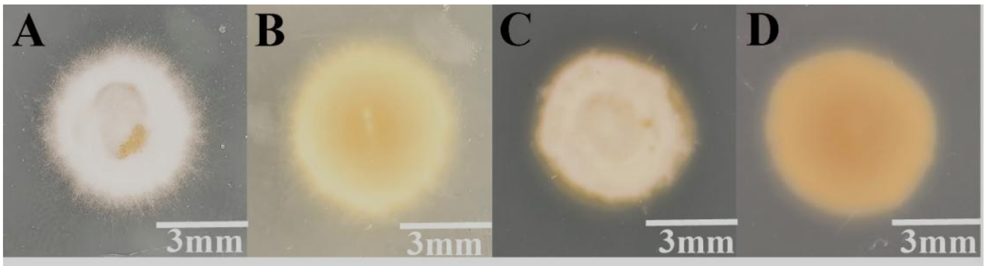

Supplementary Figure S2. Surface and reverse characteristics of fungi colonies in some strains of T2 subclass. Note: A is surface of ba31, B is reverse of ba31, C is surface of 35-4, and D is reverse of 35-4.

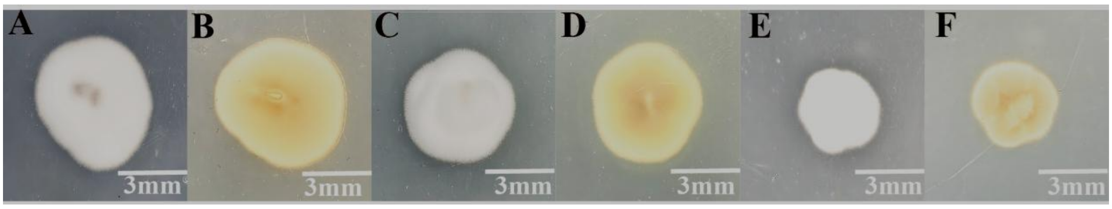

Supplementary Figure S3. Surface and reverse of fungi colonies in some strains of T3 subclass. Note: A is surface of zi4T, B is reverse of zi4T, C is surface of gan7, D is reverse of gan7, E is surface of S40, and F is reverse of S40.

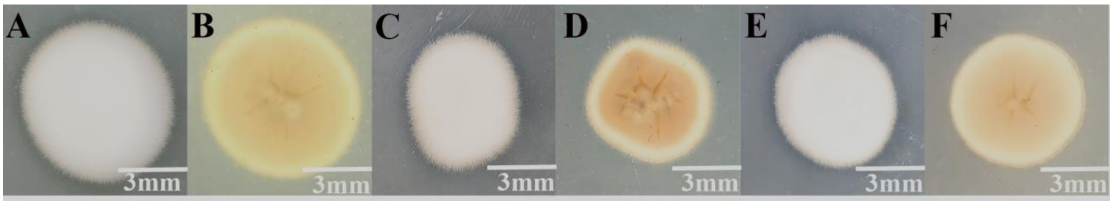

Supplementary Figure S4. Surface and reverse characteristics of fungi colonies in some strains of T4 subclass. Note: A is surface of 45-2, B is reverse of 45-2, C is surface of 20-2, D is reverse of 20-2, E is surface of gan5, and F is reverse of gan5.

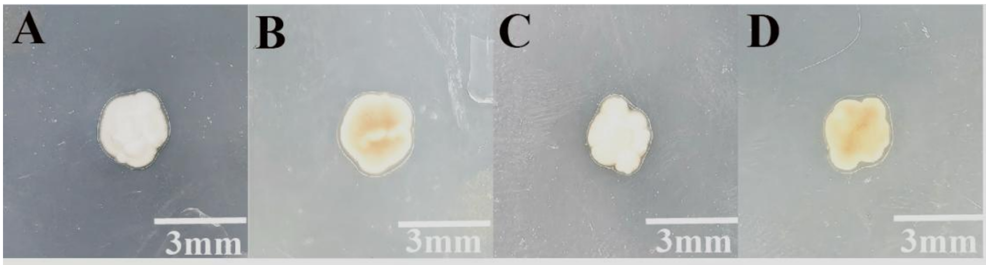

Supplementary Figure S5. Surface and reverse of fungi colonies in some strains of T5 subclass.Note: A is surface of ba24, B is reverse of ba24, C is surface of guo10T, and D is reverse of guo10T.

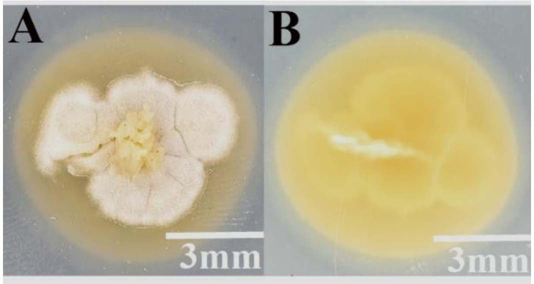

Supplementary Figure S6. Surface and reverse characteristics of fungi colonies in some strains of T6 subclass. Note: A is surface of guo4T, and B is reverse of guo4T.

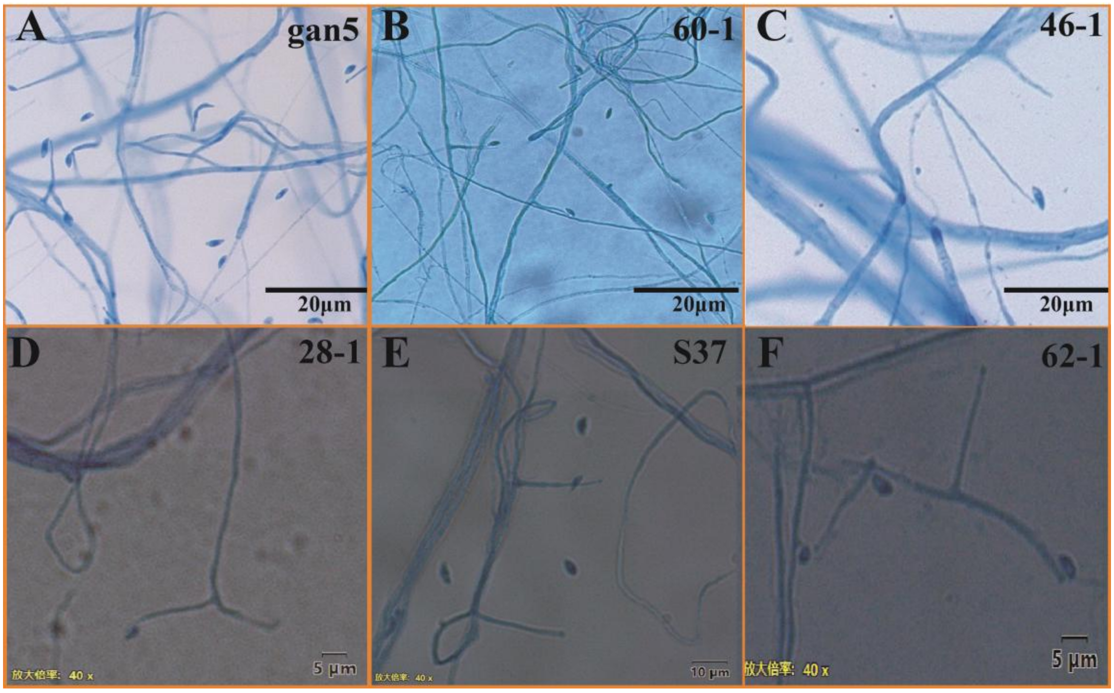

Supplementary Figure S7. The morphology of conidia and conidiophores of some *E. sinensis.* Note: A is strain gan5, with a half moon shaped spore morphology, belonging to the S1 subclass. B is strain 60-1, with elliptical spore morphology, belonging to the S2 subclass. C is strain 46-1, with spore morphology of crescent or oval, belonging to the S3 subclass. D is strain 28-1, with a half moon shaped spore morphology, belonging to the S1 subclass. E is strain S37, with elliptical spore morphology, belonging to the S2 subclass. F is strain 62-1, with spore morphology of crescent or oval, belonging to the S3 subclass.

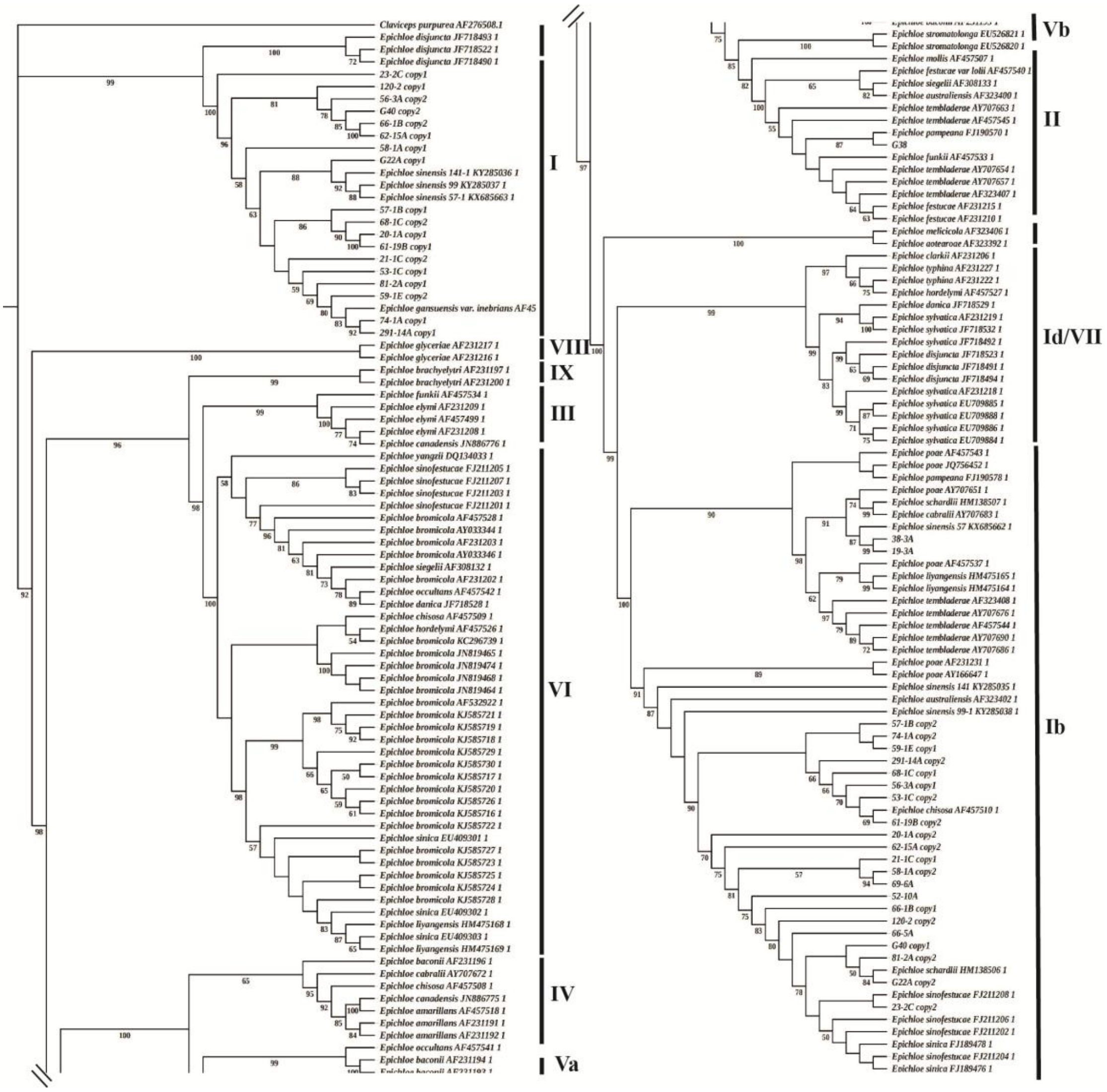

Supplementary Figure S8. Maximum likelihood phylogenetic tree constructed based on partial sequence of *tef* gene
